# Evaluation of Anionic and Cationic Pulp-Based Flocculants With Diverse Lignin Contents for Application in Effluent Treatment From the Textile Industry: Flocculation Monitoring

**DOI:** 10.3389/fchem.2020.00005

**Published:** 2020-01-30

**Authors:** Kinga Grenda, José A. F. Gamelas, Julien Arnold, Olivier J. Cayre, Maria G. Rasteiro

**Affiliations:** ^1^Department of Chemical Engineering, CIEPQPF—Chemical Process Engineering and Forest Products Research Centre University of Coimbra, Coimbra, Portugal; ^2^AQUA+TECH Specialities, Chemin du Chalet-du-Bac 4, Geneva, Switzerland; ^3^School of Chemical and Process Engineering, University of Leeds, Leeds, United Kingdom

**Keywords:** anionic cellulose, cationic cellulose, bio-polyelectrolytes, flocculation, textile wastewater, decolouration, wood wastes valorization, laser diffraction spectroscopy

## Abstract

In wastewater treatment, flocculation is a widely used solid/liquid separation technique, which typically employs a charged polymer, a polyelectrolyte (PEL). Polyelectrolytes features, such as charge type, charge density and molecular weight, are essential parameters affecting the mechanism of flocculation and subsequent floc sedimentation. The effectiveness of the process is also influenced by the characteristics of the system (e.g., type, size, and available surface area of suspended particles, pH of the medium, charge of suspended particles). Thus, a good understanding of the flocculation kinetics, involved mechanisms and flocs structure is essential in identifying the most adequate treatment conditions, having also into consideration possible subsequent treatments. In this study, *Eucalyptus* bleached pulp and a cellulosic pulp with high lignin content (~4.5 wt%) obtained from *Eucalyptus* wood waste were used for bio-PELs production. Firstly, a pre-treatment with sodium periodate increased the pulps reactivity. To produce cationic cellulose the oxidation step was followed by the introduction of cationic groups in the cellulose chains, through reaction with Girard's reagent T. Applying different molar ratios (0.975 and 3.9) of Girard's reagent T to aldehyde groups led to cationic PELs with diverse charge density. On the other hand, to obtain anionic cellulose a sulfonation reaction with sodium metabisulfite was applied to the intermediate dialdehyde cellulose-based products, during 24 or 72 h, and anionic-PELs with diverse features were obtained. The developed water soluble, anionic and cationic bio-PELs were characterized and tested as flocculation agents for a textile industry effluent treatment. Initially, jar-tests were used to tune the most effective flocculation procedure (pH, flocculant dosage, etc.). Flocculation using these conditions was then monitored continuously, over time, using laser diffraction spectroscopy (LDS). Due to the small size of the dyes molecules, a dual system with an inorganic complexation agent (bentonite) was essential for effective decolouration of the effluent. Performance in the treatment was monitored first by turbidity removal evaluation (75–88% with cationic-PELs, 75–81% with anionic-PELs) and COD reduction evaluation (79–81% with cationic-PELs, 63–77% with anionic-PELs) in the jar tests. Additionally, the evolution of flocs characteristics (structure and size) during their growth and the flocculation kinetics, were studied using the LDS technique, applying the different PELs produced and for a range of PEL concentration. The results obtained through this monitoring procedure allowed to discuss the possible flocculation mechanisms involved in the process. The results obtained with the bio-PELs were compared with those obtained using synthetic PELs, commonly applied in effluents treatment, polyacrylamides. The developed bio-PELs can be competitive, eco-friendly flocculation agents for effluents treatment from several industries, when compared to traditional synthetic flocculants with a significant environmental footprint. Moreover, LDS proved to be a feasible technique to monitor flocculation processes, even when a real industrial effluent is being tested.

## Introduction

Dye-containing wastewaters present several difficulties related to their treatment, due to their high chemical complexity, diverse dye structures usually with low molecular weight. Several industries, among which textile, paper or pharmaceutical are nowadays the most significant producers of this type of effluents. The struggle with removing dye contaminants from aqueous streams is real, since the direct discharge of dye wastes into natural water reservoirs forbidden by strict regulations, can significantly affect the environment (reduction of the dissolved oxygen, a change of pH, as well as blocking sunlight). Also, in effluents containing dyes it is common to find metals, salts, surfactants, sulfides or formaldehyde (Carliell et al., 1998), which are known for their high toxicity. Furthermore, discharged dyes without proper treatment are stable and remain in the environment for long periods of time (Hao et al., 2000). Bearing in mind the variation of the properties of dye containing effluents, due to the industrial process itself and dyes composition, as well as the presence of inorganic/organic-based additives used in the process (dos Santos et al., 2007), effective, economical and environmentally friendly treatments are required and in high demand.

Although very interesting approaches have been proposed for effective color removal, it is difficult to find a solution that works for the wide range of existing dyes (Hao et al., 2000). In fact, several factors (constraints) must be taken into account such as the type and concentration of dyes present, the eventual presence of other interfering substances, pH and the temperature of operation etc. In most wastewater treatment plants, coagulation and flocculation are widely used separation processes. These typically employ an oligomer or polymer of charged nature (polyelectrolyte). Polyelectrolytes features, such as charge type, density and molecular weight, are essential parameters affecting the conformation of a polymer chain in solution, which then influence the mechanism of flocculation and subsequent sedimentation/separation. The effectiveness of the process is also influenced by the characteristics of the system (e.g., type, size and available surface area of suspended particles, pH of the medium, charge of suspended particles). Due to the small size of the dyes molecules and high stability in the aqueous medium, additives to stimulate the destabilization of the colloidal mixtures and promote the agglomeration of sub-millimeter particles are required, which leads to the formation of flocs that can settle over a period of seconds to hours. Thus, a good understanding of flocculation kinetics, involved mechanisms and flocs structure is critical in identifying the most adequate treatment conditions for a given system.

In flocculation studies often, the floc settling behavior is measured in terms of the supernatant formation kinetics using a liquid dispersion optical characterization instrument (Turbiscan). The underlying principle of the technique is to measure and analyze the changes in the light transmission and backscattering by the sample in a cylindrical cell, as a function of particle movements (aggregation, creaming and/or sedimentation) (Kaombe et al., 2013). During the flocculation process particles aggregate, forming flocs, and become larger in size, strongly bounded and heavier than as individual particles. Increasing the combined mass enhances the sedimentation of the particles aggregates. By performing the measurement with Turbiscan the formation of the sediment layer is monitored over time. This methodology, however, is not suitable to evaluate flocculation mechanisms or to determine the floc features (structure or size) over time, as well as floc breakage.

Evolution with time of floc size and their structure can also be monitored by image analysis, which is considered as one of the easiest and the most direct measuring technique including image capture using a digital camera coupled to a microscope and its processing (Costa et al., 2013). However, this methodology requires preparation and manual selection/evaluation of a large number of samples (particles) over time in order to obtain data regarding flocculation kinetics. It is important to note that, the statistical representativeness of the sample must always be considered, and, to assure this, the technique is usually very time consuming.

An alternative technique to monitor flocculation processes is the focused beam reflectance microscopy (FBRM) (Blanco et al., 2002; Antunes et al., 2015), broadly used in real-time flocculation monitoring of large scale processes, as well as to screen the changes in particle population during the process (Liang et al., 2015). FBRM is a technique based on light scattering that does not involve sampling procedures. FBRM uses a highly focused laser beam, at a high movement speed, measuring the backward scattering light of a sample with suspended particles. The described technique can produce good quality data for particle size in relatively concentrated suspensions, but only for the large aggregates sizes. However, in the initial stages of the flocculation process where the particles size is relatively small, FBRM shows difficulties in detecting and recording good quality data, due to limited resolution, which is the main disadvantage of this technique.

Alternatively, the evolution of flocs characteristics (structure and size) during their growth, as well as the flocculation kinetics, can be studied using laser diffraction spectroscopy (LDS) (Rasteiro et al., 2007, 2008). Typically, with no particles in the system, the laser beam passes through the sample without changing its direction. The opposite happens when there are particles on the laser path. This event results in the scattering of light and alteration of the laser beam. In LDS, the angle between the scattered light and the incident beam (scattering angle) is related with the particle size. Moreover, the intensity of the scattered light is related to the number of particles in each size class. From this acquisition it is then possible to generate the scattering matrix, based on data (intensity of scattered light) for the different scattering angles, and, afterwards, particle size and size distribution can be obtained by applying an adequate model (Liang et al., 2015).

When studying the flocculation kinetics, it is very important to take into account the events of floc breakage during the process (this is not possible to observe in a typical/classical jar-test), as well as the size distribution of particles and aggregates (Rasteiro et al., 2007). Several reports refer the use of LDS in flocculation processes monitoring, in general model particle systems (Rasteiro et al., 2008). The LDS was also applied in flocculation monitoring in papermaking processes, using a standard paper filler, precipitated calcium carbonate (Rasteiro et al., 2008). Another study refers the possibility to evaluate the flocculation mechanisms involved, while working with several different synthetic flocculation agents applied in the treatment of an industrial potato crisps manufacturing effluent (Lourenço et al., 2018). The use of laser diffraction spectroscopy allowed to extract data on floc size distribution, average floc size and aggregates structure, described by the fractal dimension (dF- to characterize the primary aggregates compactness) and scattering exponent (SE- to characterize the secondary aggregates structure), in a continuous flocculation process (Rasteiro et al., 2011). The density (compactness) of the flocs can be estimated based on the fractal dimension, since it is related to the number of primary particles that fill the space in the nominal volume of an aggregate (Chakraborti et al., 2003). Furthermore, for the secondary aggregates, resulting from aggregation of the primary ones, the scattering exponent provides information for the larger length scales of the floc and their structure, as for these larger aggregates the fractal approximation is not valid (Rasteiro et al., 2011). Additionally, when using LDS it is possible to control the hydrodynamic conditions in the system (mechanical stirring rate and flow rate) so that flocculation can be conducted in controlled hydrodynamic conditions which can easily be reproduced in industrial flocculation processes (Antunes et al., 2015).

Typically, colored wastewaters from textile industries, due to their complex composition, may affect the performance of polyelectrolytes. Moreover, traditional colored water treatments, using metal-based coagulants or synthetic polyelectrolytes generate large amounts of sludge. The transport of this sludge for disposal, is the main cost and environmental burden. Therefore, the minimization of sludge production is important. A biodegradable coagulant or flocculant could reduce the volume of sludge generated. In this perspective the development and the application of novel cationic and anionic natural-based polyelectrolytes as flocculants to treat industrial effluents is of high interest (Lee et al., 2014).

The high biodegradability, and low or non-toxicity, as well as wide availability make cellulose-based PELs a more environmentally friendly option. Moreover, valorization of cellulosic wastes itself to provide end products with higher added value is also an important aspect (Grenda et al., 2019). Due to the low water solubility of cellulose it is essential to introduce charged groups, either cationic or anionic, in the cellulose backbone, and modification possibilities are limited. Pre-modification to dialdehyde cellulose, due to its efficiency (Ramírez et al., 2006) is typically applied. This reaction provides a significant modification degree, since two highly reactive aldehyde groups are introduced per anhydroglucose unit (AGU) at C-2 and C-3 positions, by opening the AGU unit at C2-C3 linkage, which allows to obtain highly modified end products—the dialdehyde cellulose (DAC). Usually selective oxidation with periodate has been applied as the first step, in which the cellulose crystalline structure is partially destroyed, associated with a decrease of the polymerization degree (Kim et al., 2000; Liu et al., 2012). Several reaction parameters may influence the properties of obtained DAC, as was reported previously (Nikolic et al., 2010; Sirviö et al., 2011a; Liu et al., 2012), which may affect further steps of the modification. Cellulose-based PELs, can be produced by introduction of positively charged groups through the cationization reaction of DAC with Girard's reagent T (Liimatainen et al., 2011; Sirviö et al., 2011b). In this procedure, more than one cationic quaternary ammonium group per AGU unit is introduced into the cellulose backbone by the formation of an imine bond. This treatment allows the end material to be highly charged (high degree of substitution) making it easy to solubilize in water at room temperature. Sirviö et al. (2011b) and Liimatainen et al. (2011) reported the modification of bleached pulp from birch wood, using the aforementioned procedure, and Grenda et al. (2019, 2020) reported the modification of bleached and unbleached *Eucalyptus globulus* pulp using the same strategy.

Typically, cellulose functionalization through anionization occurs by the introduction of sulfonate groups (-SO_3_-) in the cellulosic chain. This modification can follow the direct sulfonation of cellulose in N,N-dimethylformamide (Zhu et al., 2014) or can be based in the anionization of DAC with e.g., sodium metabisulfite in aqueous medium. The latter procedure can provide anionic cellulose-based PELs with higher ionic character, due to the substitution of more than one anionic sulfonate group per AGU unit, which significantly increases the degree of substitution and provides water solubility at room temperature. The characteristics of the anionic derivatives of dialdehyde cellulose, as the sulfonate groups content, affect their solubility (Rajalaxmi et al., 2010) what is also driven by the aldehyde content of the previously produced dialdehyde cellulose. Similar paths were described in different studies developed by Liimatainen et al. (2012) (using bleached birch (*Betula verrucosa*) chemical pulp), Hou et al. (2007) (using bleached softwood kraft pulp) or Rajalaxmi et al. (2010) (using bleached hardwood kraft pulp).

In the present study, both cationic and anionic cellulosic polyelectrolytes were produced. The reaction conditions were adjusted to obtain bio-PELs with different charges (degree of substitution). Jar-test was used to adjust the most efficient flocculation procedures, while LDS was used to evaluate continuously the flocculation process of a real industrial colored wastewater from textile industry. In all trials performed, dual systems with bentonite and cellulose-based PELs were evaluated. Bentonite promotes adsorption of the dye and the PELs act as flocculation agent of the bentonite particles with adsorbed dye. The industrial colored effluent was treated with two cationic and two anionic polyelectrolytes obtained from *Eucalyptus* bleached fibers, and with two cationic and two anionic polyelectrolytes obtained from a pulp with high lignin content (kappa number of 26.7). The size and structure of the flocs produced were monitored and analyzed, and the possible flocculation mechanisms are discussed, for each polyelectrolyte, separately. The effect on the performance during flocculation, of the chemical heterogeneity of the raw material used in the production of the bio-PELs, as well as of the degree of substitution and zeta potential of the bio-PELs, was evaluated at different pH values and flocculant concentrations. Overall, results of the assessment of cellulose-based PELs as flocculants for the treatment of a real dye-containing effluent and the tracking of the corresponding flocculation performance through LDS are presented here for the first time.

## Materials and Methods

### Raw Materials

*Eucalyptus globulus* industrial bleached kraft pulp (C_p_) (supplied by *The Navigator* Company, Portugal) was used as a reference cellulose sample of very low lignin content. The chemical composition of this pulp is summarized in Table S1.

*Eucalyptus globulus* industrial wood chips wastes, with a high size heterogeneity, supplied by *The Navigator Company* (Portugal), were used as lignocellulosic raw material. These were further processed by mild kraft pulping, in a rotary digester equipped with 4 independent 1.5 L vessels supplied by Apineq. The cooking was carried out using an active alkali charge of 14% (aqueous liquor of sodium hydroxide, sodium sulfide and sodium carbonate) and a liquor-to-wood ratio of 3.5. The reactor maximum temperature was 160 °C and the time at maximum temperature was 60 min. At the end of cooking, the produced pulp was thoroughly washed with a large amount of water and then dried. Kappa number of the final pulp was measured according to TAPPI Standard T236 om-99. The kraft pulp from wood chips wastes (C_w_) was also analyzed for sugars and lignin content. Klason and acid-soluble lignin were determined using the TAPPI Standards T 222 om-98 and T UM 250, respectively, while the sugars content was determined in the hydrolysates using high-performance liquid chromatography—HPLC. The HPLC analysis was run in an equipment from Knauer (Berlin, Germany), equipped with a Smartline pump 1000, Smartline RI Detector S2300 and an Agilent Hi-Plex Ca, 300 x 7.7 mm column from Agilent Technologies. The results of the chemical analysis of C_w_ pulp are presented in Table S1.

### Modification of Pulps and Characterization of the Obtained Materials

#### Pulp Oxidation Pre-treatment by Reaction With Sodium Periodate

The periodate oxidation of *Eucalyptus globulus*-based pulps (C_p_ and C_w_) followed the procedure described elsewhere (Grenda et al., 2017). In general, prior to the process, 4 g (dry basis) of pulp (C_p_ or C_w_) were disintegrated/swelled overnight in distilled water at 4% consistency. The suspension was then treated with a mixture of 300 mL of distilled water, 7.2 g of LiCl and 8.2 g of NaIO_4_. A highly oxidized material was produced after 3 h of oxidation at 75°C, which was filtered and washed with distilled water. The non-dried cellulose-based dialdehyde (DAC_p_ or DAC_w_) product was stored in the fridge and used later for further modifications (cationization and anionization). Moreover, DAC samples oven-dried at 60°C were used for FTIR-ATR measurements. The aldehyde content of DAC was determined based on the oxime reaction between aldehyde groups and NH_2_OH·HCl, as described in the literature (Grenda et al., 2017), having been obtained the values of 10.96 mmol aldehyde/g for DAC_p_ and 10.19 mmol aldehyde/g for DAC_w_.

#### Preparation of Water-Soluble Cationic Cellulose-Based Polyelectrolytes

Cationization of non-dried DAC_p_ or DAC_w_ was performed through reaction with Girard's reagent T (GT) at two different GT/aldehyde molar ratios, 0.975 and 3.9, at pH 4.5 in 80 mL of distilled water. The reaction mixture was stirred for 1 h at 70°C for cationization to occur. After cooling, isopropanol was added to precipitate the soluble product (CDAC—cationic dialdehyde cellulose-based polyelectrolyte). The mixture was centrifuged for 30 min at 4,500 rpm, after which, the supernatant was removed. The solid product was washed several times with a water/isopropanol solution (1/9, v/v), until the supernatant showed no GT. Removal of the residual GT was monitored by adding a small amount of AgNO_3_ to the supernatant (absence of AgCl precipitate indicated washing was complete). Applying two different GT/aldehyde molar ratios allowed to obtain cationic cellulose-based polyelectrolytes with diverse characteristics. The final cationic products were oven-dried at 60°C and then stored in a desiccator for further characterizations and evaluation as flocculation agents.

#### Preparation of Water-Soluble Anionic Cellulose-Based Polyelectrolytes

The anionization of non-dried DACs, either DAC_p_ or DAC_w_ was performed through reaction of 14 mmol of sodium metabisulfite/g DAC in 60 mL of deionized water. The reaction mixture was stirred with a magnetic stirrer for 24 or 72 h at room temperature (25°C). The flask was well sealed during the reaction to avoid any changes of concentration. After reaction, the transparent solution was also mixed with isopropanol to precipitate the soluble product, and then the mixture was centrifuged at 4,500 rpm for 45 min. The separated solid was then washed with a water/isopropanol solution (1/9, v/v). The anionic DAC (ADAC) product was oven-dried at 60°C and stored in a desiccator. Through application of different anionization times (24 and 72 h), anionic cellulose-based products with varied degrees of anionization were obtained. The final anionic celluloses were characterized by several techniques and further used as flocculation agents.

#### Characterization of Water-Soluble Cationic and Anionic Cellulose-Based Polyelectrolytes

The obtained cationic and anionic cellulose-based PELs were characterized by FTIR spectroscopy to confirm the presence of the new functional groups introduced in the polysaccharide's backbone (Grenda et al., 2019) and by elemental analysis in order to determine the cationicity or anionicity index of the products. The nitrogen content assessed by elemental analysis was used to obtain the degree of cationization while the sulfur content was used to determine the degree of anionization of cellulose in the ADAC samples. The results obtained (N% or S%, w/w) were an average of at least 3 measurements.

Hydrodynamic diameter and zeta potential measurements of the PELs were also performed, by dynamic light scattering (DLS) and electrophoretic light scattering (ELS), respectively, in a Zetasizer NanoZS, ZEN3600, from Malvern Instruments, with temperature set up to 25°C. For the hydrodynamic diameter, backscatter detection at 173° angle was employed. Using automatic measurements mode, with at least 5 repetitions of the measurement, the average hydrodynamic diameter (nm) (z-average diameter) and the PDI (polydispersity index) of the hydrodynamic diameter distribution were obtained. Similarly, zeta potential values were taken as an average of 5 repeated measurements. Details of the sample's preparations can be found elsewhere (Grenda et al., 2019).

### Evaluation of Performance in Textile Wastewater Treatment

#### Industrial Effluent Characterization

In the present study, a colored industrial effluent was supplied by *Rosarios4*, Portugal. The textile wastewater was collected from a single dyeing session, and contained only one shade of Turquoise Blue dye. The effluent characterization in terms of COD, pH, turbidity and zeta potential, is summarized in Table S2.

The zeta potential of the initial industrial colored effluent as well as the changes in zeta potential as a function of pH were measured using Electrophoretic Light Scattering (ELS) in a Malvern Zetasizer Nano ZS, model ZEN3600 (Malvern Instruments Ltd, UK). At desired pH 1 mL of sample was injected directly into the disposable plastic capillary cell and the measurements were conducted at 25°C, using the automatic measurement mode, with at least 5 repetitions of the measurement.

#### Flocculation Experiments

Jar-tests were applied in order to pre-evaluate the flocculation performance of the water-soluble cationic (CDAC_p_ and CDAC_w_) and anionic (ADAC_p_ and ADAC_w_) cellulose-based polyelectrolytes with diverse chemical complexity (lignin contents) that possessed two different degrees of substitution of charged groups (either cationic or anionic). The cellulose-based polyelectrolyte solutions used in the flocculation tests were prepared by dissolving the CDAC or ADAC, at 0.1 wt% concentration, in distilled water, and stirring at 500 rpm for 30 min.

For the flocculation experiment (jar-test), a volume of 100 mL of pre-agitated colored effluent, at room temperature, was adjusted to the required pH using hydrochloric acid (HCl) aqueous solution, with a pH meter SCAN3BW (Scansci). Three different pH values (1.5, 3.0, and 7.0) were considered to assess the influence of pH on the flocculation performance. A suitable dosage of flocculant was then added dropwise to the effluent, while mixing slowly during 20 s. For most of the experiments, bentonite purchased from Vermeer Portugal under the commercial name Cebo Premium (which possessed ζ-potential of −43 ± 1 mV, d(0.5) - median of the particle size distribution of 2.3 μm and D[3.2] - surface weighted mean of the particle size distribution of 2.1 μm) was also added before the flocculant addition. Supernatant samples of approximately 5 mL were collected at about 75% height from the center of the beaker, for evaluation of the effluent clarification over time (1 min, 30 min, 1 h, and 24 h). The color removal (clarification) was calculated based on Equation 1, by measuring the initial turbidity of the effluent and the turbidity of the supernatant after a certain settling time.

(1) $$\begin{array}{r} Color\;removal\;\left(\%\right)=\frac{T_{0}-T_{f}}{T_{0}}\cdot 100 \end{array}$$

where T_0_ is the turbidity of the initial wastewater at time zero, and T_f_ is the turbidity of the supernatant of the treated sample at a given time. In some cases, values of turbidity removal lower than zero were obtained. This is because just after addition of bentonite turbidity usually increases, and, if the settling of the particles is poor, even after further addition of flocculant, for non-optimal conditions turbidity can still be higher than that of the original effluent, providing thus negative values of color removal.

At least three repetitions of the turbidity measurements of each sample, using a Photometer MD600 (Lovibond, UK), were performed. The variation between replica of turbidity measurements for each sample was always below 1%. Comparison of the efficiency of the new bio-PELs on color removal, was made with commercial cationic (cPAM- SnowFlake E2 with 45 wt% charge) and anionic (aPAM- SnowFlake X0 with 30 wt% charge) polyacrylamides of similar charge, supplied by aquaTECH, Geneva.

The chemical oxygen demand (COD) of the treated supernatant was measured for selected trials (polymers and procedures) after 24 h of settling. COD tests were performed with 2 mL of supernatant, added directly to the COD test tube (COD Kit, Lovibond, UK) and allowed to remain at 150°C for 2 h in the thermo-reactor (VELP Scientifica). After reaching room temperature, and resting for 12 h in dark conditions, the COD value was measured in the Photometer MD600 (Lovibond, UK).

Additionally, laser diffraction spectroscopy (LDS), Malvern Mastersizer 2000 (Malvern Instruments), was used to monitor the flocculation process in low mixing conditions, following the procedure developed previously by the authors (Lourenço et al., 2018). LDS was used to evaluate the flocculation performance of modified cellulose from bleached pulp both cationized (CDAC_p_A, B) and after introducing anionic groups (ADAC_p_A, B), as well as modified cellulose obtained from a pulp with a kappa number of 26.7, either with cationic (CDAC_w_A, B) or anionic (ADAC_w_A, B) groups. To conduct the LDS tests a 50% dilution with distilled water, of the initial industrial effluent used in the jar-tests, was required, to ensure an acceptable level of obscuration in the equipment, after addition of the appropriate amount of the complexation agent (bentonite). This is the main limitation when using LDS to monitor flocculation processes, even if in most cases results obtained follow the same trends obtained in the jar-tests. The concentrations of the used treatment agents were recalculated considering the dilution factor, to reproduce the procedures evaluated in the jar-tests. Initial turbidity of the effluent with bentonite, before dilution, was 212 NTU (177 NTU effluent initial turbidity), while after dilution it was reduced till 100 NTU. A volume of 23 mL of bentonite suspension (at 5 wt%) was added to 750 mL of the effluent in the equipment dispersion unit, which corresponded to an average bentonite concentration of 0.15 wt%. The initial industrial effluent, at pH 3.0, was stirred at a speed of 2,000 rpm to guarantee homogenization, and the size distribution of the particles in the effluent was acquired. Bentonite was then added at 1,200 rpm, followed by the addition of the cellulose-based PELs, using the same stirring speed. The overall concentrations of PEL in the system tested were: 1.3, 2.6 mg/l (corresponding to procedure A and D in the jar-tests), 4.0 and 5.3 mg/L (corresponding to procedure B and C in the jar-tests). During the monitoring of the flocculation process the tests were carried out at the optimized stirring speed of 350 rpm (shear rate 20s^−1^ based on CFD modeling of the sampling vessel) in the flocculation vessel of the Malvern Mastersizer 2000, to guarantee an effective floc circulation in the sampling system, ensuring that floc sedimentation was not occurring and avoiding also floc breakage. The size of the flocs was measured every 36 s for a total period of 20 min (i.e., until the floc size stabilized).

The scattering matrix obtained by LDS was treated off-line to calculate the scattering exponent (SE) of the flocs (Rasteiro et al., 2011). The SE parameter provides information about floc structure. From the scattering matrix obtained by LDS it is possible to plot, in logarithmic scale, the scattering intensity vs. q (see Equation 2), and the slope of the first region of this plot is related to SE (Rasteiro et al., 2011). The scattering matrix, acquired during each measurement, can be exported through the equipment software and then processed, offline, for each acquisition, in order to obtain the scattering exponents, including their evolution over time.

(2) $$\begin{array}{r} q=\frac{4\pi {\;n}_{0}}{\lambda_{0}}\sin\left(\theta /2\right) \end{array}$$

Where n_0_ is the refractive index of the dispersing medium, θ is the scattering angle and λ_0_ corresponds to the incident light wavelength.

## Results and Discussion

### Synthesis and Characterization of Cationic and Anionic Cellulose-Based Polyelectrolytes

**Figure 1:**
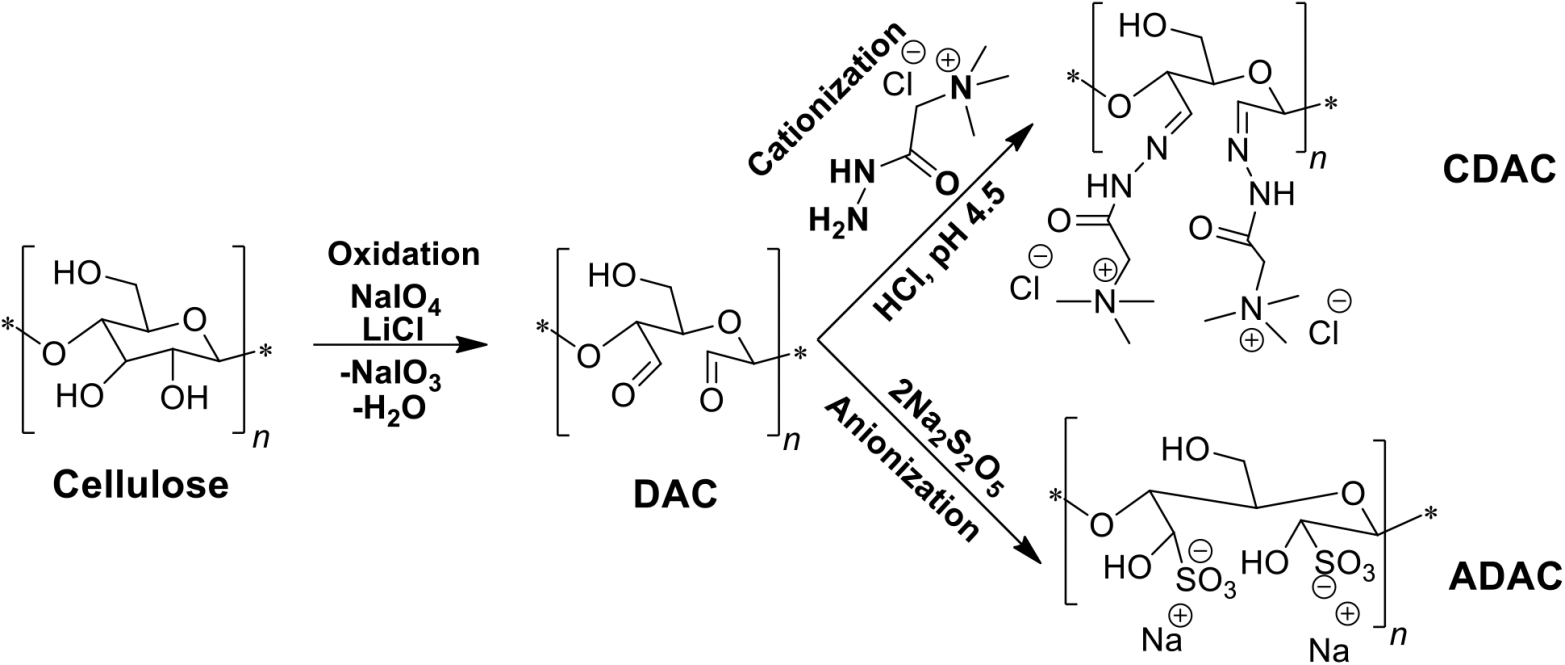
Production of positively and negatively charged cellulose-based PELs, by periodate oxidation (DAC) followed by cationization of DAC with Girard's reagent T (CDAC) or anionization with sodium metabisulfite (ADAC).

Derivatization of different *Eucalyptus*-based cellulosic materials (see Table S1), in order to obtain cationic or anionic PELs, was carried out following the route described in Figure 1. Initially, DAC was produced by periodate oxidation of the cellulose-rich material. The resultant DAC was reacted with Girard's reagent T yielding the cationic derivative (CDAC), or with sodium metabisulfite producing the anionic derivative (ADAC).

The periodate oxidation as well as the quaternary ammonium cationization procedure with Girard's reagent T developed for *Eucalyptus* bleached fibers, described by Grenda et al. (2020), was implemented in the same way for a pulp with high lignin content (4.4%, kappa number of 26.7) obtained from Eucalyptus wood wastes. It is expected that different composition of the raw materials, namely the cellulose, hemicellulose (xylan) and lignin content can influence not only the characteristics of the final polyelectrolytes but also their potential application as flocculants. Applying different GT/aldehyde molar ratios allows to obtain different degrees of substitution by the cationic unit (see Table 1). There is a trend of decrease of zeta potential of the final product, with the increase of complexity of the raw material used in the periodate oxidation, and the lowest value was observed while performing cationization of the wood pulp with a high kappa number of 26.7 at low GT/aldehyde molar ratio. Higher complexity of the raw material, namely larger lignin and xylan content, led to obtain cationic cellulose-based PELs with lower zeta potential that varied from 40 ± 3 mV (CDAC_w_B; obtained using 0.975 of GT/aldehyde molar ratio) to 46 ± 1 mV (CDAC_w_A; using 3.9 GT/aldehyde molar ratio). While applying the same conditions to the pulp with higher chemical homogeneity (bleached pulp, negligible lignin content) the obtained polyelectrolytes were characterized by a higher positive charge (zeta potential of 52–54 mV). However, the obtained CDAC_p_ products presented significantly reduced hydrodynamic diameter when compared to the CDAC_w_ products, probably due to a reduction of the cellulose chain length during bleaching.

**Table 1:** Cationization reaction conditions used for synthesis of cationic bio-PELs and characterization of final products.

**Name**	**Time (h)**	**Temp (^**°**^C)**	**GT/aldehyde (molar ratio)**	**Cationicity index (mmol/g)**	**ζ-potential (mV)**	**Z-Average diameter (nm)**	**PDI**
CDAC_p_A	1	70	3.9	3.74	52 ± 2	124 ± 2	0.34 ± 0.03
CDAC_p_B	1	70	0.975	3.08	54 ± 1	176 ± 4	0.5 ± 0.01
CDAC_w_A	1	70	3.9	3.56	46 ± 1	247 ± 8	0.43 ± 0.02
CDAC_w_B	1	70	0.975	2.84	40 ± 3	292 ± 10	0.45 ± 0.02

*Cationicity determined as the amount of alkylammonium groups (mmol) per g (dry weight) of cationic cellulosic sample; PDI- polydispersity index of the hydrodynamic diameter distribution; cationic cellulose-based polyelectrolytes from bleached pulp or from wood wastes pulp with kappa number 26.7, respectively, CDAC_p_ and CDAC_w_*.

The synthesis of anionic cellulose-based polyelectrolytes (ADAC) followed the reaction described in Figure 1. Table 2 shows the reaction conditions used to produce the ADAC samples and the characterization results. Again, different initial materials were considered (*Eucalyptus* bleached pulp and a pulp with high lignin content). The reaction time was varied from 24 to 72 h, while keeping constant the reaction temperature (room temperature, 25°C) and the molar ratio of sodium metabisulfite to the aldehyde groups' content of DAC. Once more, it is expected that different composition of initial raw materials used in the production of DAC precursors for anionization, namely a lower content of cellulose and the presence of other constituents as lignin, should have some influence not only on the reaction kinetics but also on the characteristics of the obtained ADACs. The influence of the different reaction times (24–72 h) on the characteristics of the final products (anionic groups content, zeta potential, hydrodynamic diameter and PDI) is summarized in Table 2. Again, when using a more heterogeneous starting material, the zeta potential of the modified cellulose-based PELs is lower. It was also observed that with the increase of reaction time there is a slight decrease in the hydrodynamic diameter of the obtained anionized products, independently of the raw material used. Regarding the degree of substitution by anionic groups there is a clear trend of the effect of reaction time in the case of the ADAC_w_ products.

**Table 2:** Anionization reaction conditions used for synthesis of anionic bio-PELs and characterization of final products.

**Name**	**Time (h)**	**Temp (^**°**^C)**	**Anionicity index (mmol/g))**	**ζ-potential (mV)**	**Z-Average diameter (nm)**	**PDI**
ADAC_p_A	24	25	4.23	−44 ± 2	157 ± 10	0.59 ± 0.08
ADAC_p_B	72	25	4.17	−50 ± 1	127 ± 6	0.52 ± 0.02
ADAC_w_A	24	25	4.47	−45 ± 1	137 ± 8	0.47 ± 0.02
ADAC_w_B	72	25	4.90	−36 ± 2	116 ± 7	0.46 ± 0.03

*Anionicity determined as the amount of sulfonate groups (mmol) per g (dry weight) of anionic cellulosic sample; PDI- polydispersity index of the hydrodynamic diameter distribution; anionic cellulose-based polyelectrolytes from bleached pulp or from wood wastes pulp with kappa number 26.7, respectively, ADAC_p_ and ADAC_w_*.

The solubility of CDACs and ADACs was confirmed by the total transparency of the reaction solution at the end of the modifications. The overall results showed that highly charged, cellulose-based cationic and anionic polyelectrolytes can be prepared by a two-step modification: periodate oxidation/cationization or periodate oxidation/anionization sequence, using various GT/aldehyde molar ratios (in the case of cationization) or different reaction times (while performing anionization). As a result, a range of PELs with different charge densities were prepared that apparently exhibit adequate features to be applied as flocculation agents in industrial wastewater treatment. The final evaluation of the products produced (cellulosic PELs) is going to be conducted by jar-test as well as by LDS.

### Evaluation of Performance of Cellulosic Pels in Textile Wastewater Treatment

The performance of obtained cationic (CDAC_p_A, B and CDAC_w_A, B) and anionic (ADAC_p_A, B and ADAC_w_A, B) cellulose-based polyelectrolytes (with diverse characteristics) in the decolouration of an effluent from textile industry will be presented here and discussed individually, for each type of cellulosic polyelectrolyte (cationic or anionic). Also, the influence of the source of the raw material used in the production of tested bio-PELs, as well as their substitution degree, is going to be evaluated, based on the jar-tests and on LDS studies. In all performed jar-tests the cellulosic PELs were compared with a synthetic reference, either cationic, or anionic PAM (Grenda, 2018).

When applying flocculation or coagulation strategies in the water treatment, it is very important to understand the charge characteristics of the tested system. The stability of dye effluents can significantly influence the success of used procedure. Determination of the particles charge is then crucial and the stability of the system can be tuned by changing the pH of the system, due to ionization of certain groups in the dye structures. The zeta potential of the industrial effluent, after adjustment for the pH's to be used in the performance jar-tests, was measured, and the obtained values are presented in Figure S1. For the tested effluent, the particles surface is mainly negatively charged. With the increase of alkalinity, a significant increase in negative charge was also observed. The highly negatively charged suspended dye molecules of the system tested promotes the stabilization of the effluent, due to the repulsion effect, making it more difficult to treat the effluent at alkaline pH. It is then expected that pH changes will impact clarification performance of tested effluent and better color removals are going to be obtain at acidic conditions where the effluent presents lower charges. At neutral pH, pH 7, the zeta potential of the colored effluent tested was clearly in the stable zone (-49 mV).

#### Jar-Test Flocculation Study

Cationic cellulose-based flocculants, hold positively charged groups, which are fundamental for the neutralization of negatively charged suspended dye molecules. Also, due to the relatively long chains of the polymers with medium charge densities, bridging between the particles will be a complementary mechanism. The adsorbed polymer chain can extend from the particle surface and interact with other particles or polymer chains.

**Figure 2:**
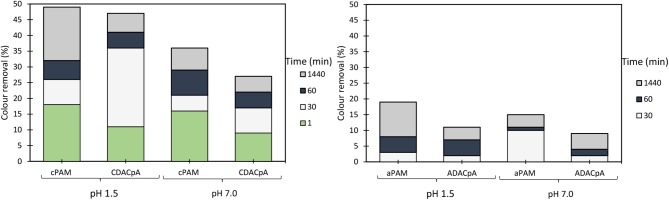
Industrial colored effluent treatment (based on turbidity reduction) using only a single flocculation agent [5.34 mg/L of synthetic (cPAM or aPAM) or cellulose-based (CDAC_p_A or ADAC_p_A)] at two different pH levels, 1.5 and 7.0.

**Figure 3:**
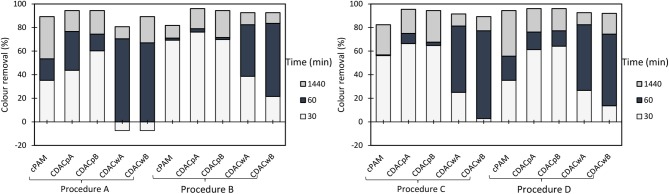
Turbidity removal of industrial effluent as a function of time of settling, using cationic cellulose-based flocculation agents from bleached pulp (CDAC_p_ A and B) and from wood chips (CDAC_w_ A and B), in a dual system with bentonite, at pH 1.5. Procedure A: 0.3 wt% bentonite followed by 2.67 mg/L of flocculant. Procedure B: 0.6 wt% bentonite and 5.34 mg/L of flocculant. Procedure C: 0.3 wt% bentonite and 5.34 mg/L of flocculant. Procedure D: 0.6 wt% bentonite and 2.67 mg/L of flocculant. A cationic synthetic cPAM (in dual system with the same concentrations described in procedures A-D), was used as reference.

**Figure 4:**
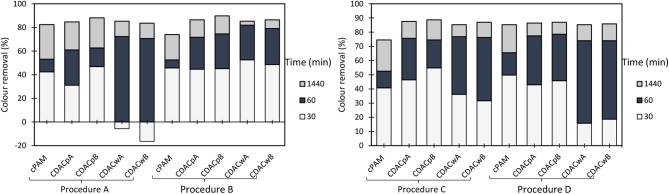
Turbidity removal of industrial effluent as a function of time of settling, using cationic cellulose-based flocculation agents from bleached pulp (CDAC_p_ A and B) and from wood chips (CDAC_w_ A and B), in a dual system with bentonite, at pH 3.0. Procedure A: 0.3 wt% bentonite followed by 2.67 mg/L of flocculant. Procedure B: 0.6 wt% bentonite and 5.34 mg/L of flocculant. Procedure C: 0.3 wt% bentonite and 5.34 mg/L of flocculant. Procedure D: 0.6 wt% bentonite and 2.67 mg/L of flocculant. A cationic synthetic cPAM (in dual system with the same concentrations described in procedures A-D), was used as reference.

**Figure 5:**
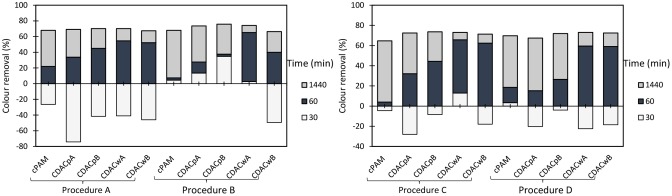
Turbidity removal of industrial effluent as a function of time of settling, using cationic cellulose-based flocculation agents from bleached pulp (CDAC_p_ A and B) and from wood chips (CDAC_w_ A and B), in a dual system with bentonite, at pH 7.0. Procedure A: 0.3 wt% bentonite followed by 2.67 mg/L of flocculant. Procedure B: 0.6 wt% bentonite and 5.34 mg/L of flocculant. Procedure C: 0.3 wt% bentonite and 5.34 mg/L of flocculant. Procedure D: 0.6 wt% bentonite and 2.67 mg/L of flocculant. A cationic synthetic cPAM (in dual system with the same concentrations described in procedures A–D), was used as reference.

The influence of pH and dosage of inorganic complexation agent (bentonite), as well as the dosage of cellulose-based flocculants obtained from *Eucalyptus*-based raw materials of different chemical complexity (cellulose and lignin contents), on the turbidity reduction and on color removal are summarized in Figures 2–5. Figure 2 presents the results obtained using a single system, only the polyelectrolyte, either synthetic, or natural-based (either anionic or cationic).

In this study, the values of the supernatant water turbidity were used to evaluate the PELs performance in the treatment of this industrial effluent. Figure 2 shows the color removal results at two different pH's (1.5 and 7.0) for 5.34 mg/L CDAC_p_A or synthetic cPAM, in single system (left insert) or when using 5.34 mg/L ADAC_p_A or synthetic aPAM, also in single system (right insert). The decolouration results were always better for acidic conditions for both cellulose-based and synthetic flocculation agents. At pH 1.5, CDAC_p_A was more effective in this effluent treatment: in the first hour of treatment it allowed to remove 36% of color (after 30 min) and 41% (after 1h), while cPAM removed 26 and 32%, respectively after 30 min and 1 h of treatment. For a long period of treatment, after 24 h of settling, both polymers showed similar color reduction. Moreover, at neutral pH (7.0) cPAM showed a slightly superior performance over CDAC_p_A, removing 36% of turbidity after 24 h, while the cellulose-based flocculant removed 27%. The better results obtained with both flocculants for acidic pH are due to the effluent lower stability (Figure S1) for this pH; the lower repulsion effect between the particles, makes it easier to treat the effluent at low pH, when compared to more alkaline pH levels. As mentioned before, also with the anionic polyelectrolytes, the best results (19% turbidity reduction after 24 h) were obtained when pH was adjusted to 1.5, due to the lower effluent stability. When comparing the performance of the synthetic aPAM to that of the natural-based ADAC_p_A, lower turbidity reductions were always obtained for the latter one, for the same pH levels, possibly due to a slightly lower charge density and molecular weight of the anionic natural-based PEL, compared to the synthetic one. As expected, considering the average negative charge of the effluent for the different pHs, the cationic PELs performed better than the anionic ones.

**Figure 6:**
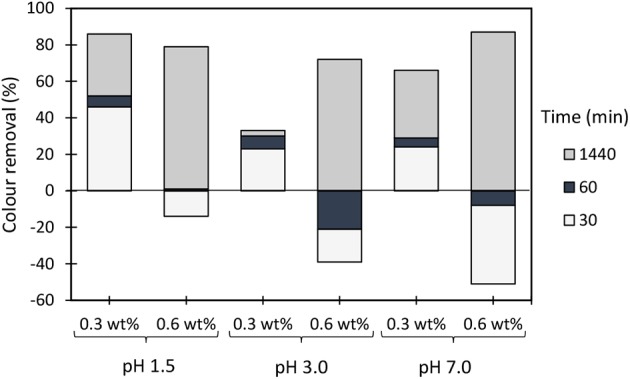
Industrial colored effluent treatment (based on turbidity reduction) when using only bentonite at two different dosages (0.3 and 0.6 wt%) and three different pH levels, 1.5, 3.0, and 7.0.

Considering that removal with single flocculation system was still not adequate (maximum around 50% for the cationic PELs at pH 1.5), the industrial effluent was also treated using a dual system with bentonite (an inorganic material). Figures 3–5 show results obtained at pH 1.5, 3.0, and 7.0 (the initial effluent pH was 12.0) while using two different concentrations of flocculation agent (either natural-based or synthetic) 2.67 mg/L (procedures A and D) or 5.34 mg/L (procedures B and C), and two different amounts of bentonite 0.3 wt% (procedures A and C) or 0.6 wt% (procedures B and D). Additionally, all procedures A - D at all tested pH levels were compared with the treatment performed only with bentonite (mono system, see Figure 6). In all the presented cases A - D and tested pH levels, better color removal was always obtained after 24 h of treatment, while using dual system (bentonite followed by addition of flocculant), when compared to procedures using only bentonite (Figure 6), or single system with flocculant (Figure 2), at the same pH.

As can be seen, in Figure 3, at pH 1.5, due to the highest instability of the treated effluent, higher turbidity removals were obtained in general, when compared to the other pHs (3.0 and 7.0) (see Figures 4, 5). Also, at high acidic pH bentonite tends to have the most positively charged surface (Kim, 2003) compared to pH 3.0 or 7.0. Thus, complexation of the dye with the particles is enhanced, allowing PELs to interact with those complexes, and then flocculate through the bridging mechanism. Moreover, with the increase of bentonite or flocculant dosage, performance did not improve significantly regarding final removal (24 h), even if when using procedure B settling was faster. Thus, at pH 1.5 both procedures A and B showed to be the most effective. Interactions should be mainly electrostatic between alkylammonium groups of cationic cellulosic PEL (Figure 1) and sulfonate groups of negatively charged dyes. Hydrogen bonding through hydroxyl groups of the cellulosic backbone and amine functions of the dye may also be effective, as well as van der Waals interactions (Lapointe and Barbeau, 2020). Initially, the interactions of the dye with bentonite should be mainly electrostatic. Bridging must then be the main flocculation mechanism of the bentonite particles after addition of the cellulose PELs. The molecular weight of the cellulose-based PELs was not determined. Notwithstanding, considering a typical polymerization degree of the initial kraft pulps used in the present work near 3000 (corresponding to a molecular weight of around 486 kDa) (Lourenço et al., 2019), and even considering that some depolymerization of cellulose has occurred during the PEL production, namely in the step of periodate oxidation, it is reasonable to expect a final PEL still with a medium molecular weight, for which bridging mechanisms are possible (Bolto and Gregory, 2007).

Additionally, for the best treatment conditions, CDAC_p_A and CDAC_p_B presented superior efficiency in the reduction of turbidity 44% (30 min), 77% (1 h), and 94% (24 h) for CDAC_p_A and 60% (30 min), 74% (1 h), and 94% (24 h) for CDAC_p_B due to their high cationicity index and substitution degree, when compared with the CDAC_w_ samples. CDAC_p_ are natural-based polyelectrolytes obtained from relatively more homogenous raw material (bleached fibers) compared to the other tested cellulose-based PELs. This trend appeared for all the pHs and conditions tested. It is worth stressing that the not so good performance of CDAC_w_ (A and B) is more obvious when referring to the rate of turbidity removal (settling is lower) than when we compare the final turbidity removal results. Comparing Figures 3, 4, it is also clear that good, but slightly lower turbidity reductions, were obtained at pH 3.0, compared to performance at pH 1.5. The worst results, especially for low settling times, even if still reasonable, were obtained for pH 7 (Figure 5). This indicates a higher stability of the effluent at this pH, which resulted in an increase of turbidity in the first moments after addition of bentonite.

Furthermore, in dual system, results obtained with the natural-based flocculants are very similar or slightly higher than the ones obtained with the synthetic reference cPAM.

**Figure 7:**
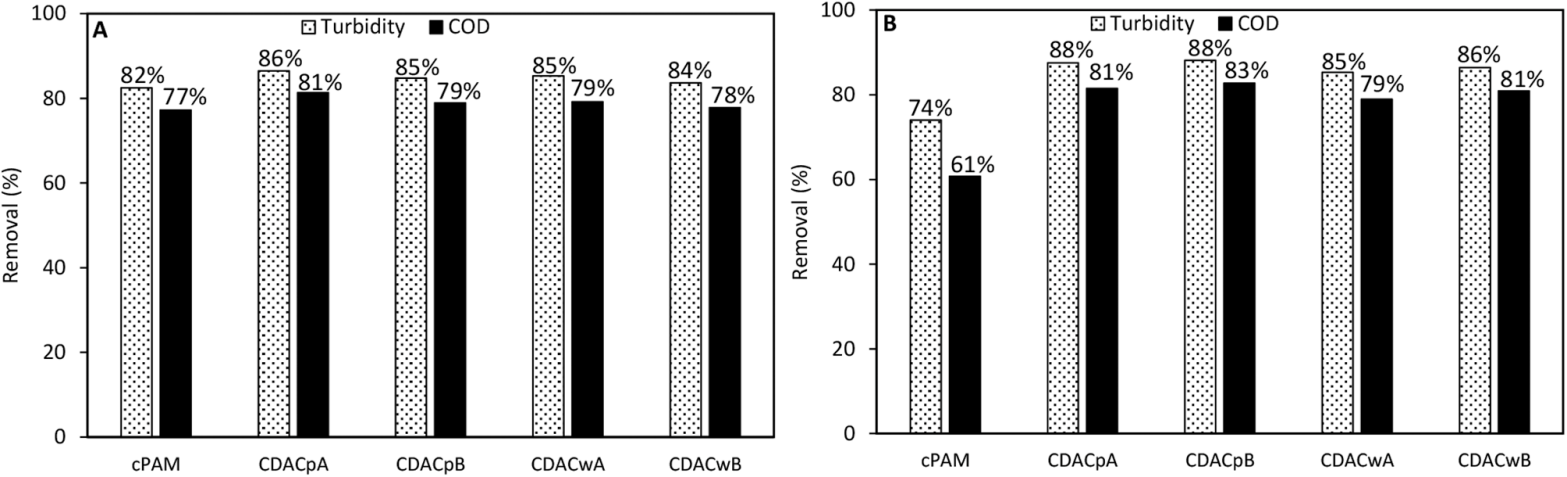
COD reduction and turbidity removal during the industrial effluent treatment with CDAC_p_A, B and CDAC_w_ A, B and reference Snow Flake E2 (cPAM), at pH 3.0, after 24 h of treatment, in two conditions: **(A)** 0.3 wt% bentonite followed by 2.67 mg/L of flocculation agent and **(B)** 0.6 wt% bentonite followed by 5.34 mg/L of flocculation agent.

For the cationic natural-based PELs from modified bleached pulp, with high (CDAC_p_A), and lower (CDAC_p_B) substitution degree, as for the cationic PELs from wood wastes, obtained from pulp with significant lignin content (kappa number 26.7), CDAC_w_A (higher cationicity index) and CDAC_w_B (lower cationicity index), and for the reference polymer cPAM (Snow Flake E2), COD reduction was measured, after 24 h of treatment, at pH 3.0, for two conditions: with minimum dosages of bentonite and flocculant, which corresponds to procedure A (0.3 wt% bentonite followed by 2.67 mg/L of flocculation agent) and with maximum dosage of the two components, procedure B (0.6 wt% bentonite followed by 5.34 mg/L of flocculation agent). The results of COD reduction together with the turbidity removal after 24 hours obtained using these procedures are plotted in Figure 7. The COD reduction is closely related with the turbidity removal, and with the increase of effluent clarification higher COD reduction was observed. For the procedure with addition of lower amounts of treatment agents (procedure A), the COD reduction obtained was in the range of 78–81%, for the new natural-based flocculation agents, while for the synthetic reference was 77%. Moreover, procedure B, with double amounts of added components, when compared to procedure A, presents only slightly higher turbidity removals and COD reductions. In this case, the COD reduction obtained was in the range of 79–83%, for the new natural-based flocculation agents, while for the synthetic reference only 61% of COD reduction was achieved. Considering the overall results, the natural-based flocculation agent CDAC_p_A, seems to be the cellulose-based flocculant that has the best performance in the treatment of the industrial colored effluent (higher turbidity reduction, with low dosage, and high COD reduction) among the tested cationic flocculation agents, leading also to faster decolouration (faster settling) (see also Figures 3–5).

On the other hand, the cationic cellulose-based flocculation agents, namely CDAC_p_A, showed superior performance in turbidity removal and COD reduction, over the synthetic cPAM reference. It is worth stressing that the use of procedure A, with low addition of bentonite (0.3 wt%), followed by a low dosage of CDAC (2.67 mg/L) showed good results, similar to the ones with increased dosages of used components, which is a positive aspect, since using lower dosage of bentonite and flocculant can significantly reduce the costs associated to the treatment process.

**Figure 8:**
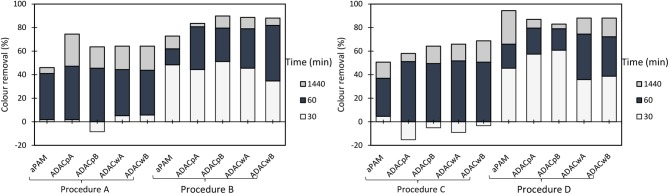
Turbidity removal of industrial effluent as a function of time of settling, using anionic cellulose-based flocculation agents from bleached pulp (ADAC_p_ A and B) and from wood chips (ADAC_w_ A and B), in a dual system with bentonite, at pH 1.5. Procedure A: 0.3 wt% bentonite followed by 2.67 mg/L of flocculant. Procedure B: 0.6 wt% bentonite and 5.34 mg/L of flocculant. Procedure C: 0.3 wt% bentonite and 5.34 mg/L of flocculant. Procedure D: 0.6 wt% bentonite and 2.67 mg/L of flocculant. An anionic synthetic aPAM (in a dual system with the same concentrations described in procedures A-D), was used as reference.

**Figure 9:**
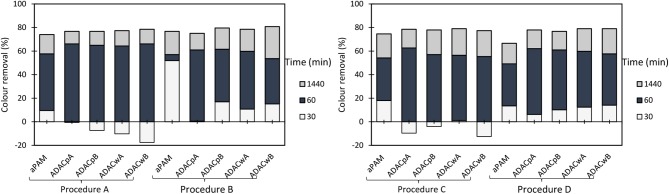
Turbidity removal of industrial effluent as a function of time of settling, using anionic cellulose-based flocculation agents from bleached pulp (ADAC_p_ A and B) and from wood chips (ADAC_w_ A and B), in a dual system with bentonite, at pH 3.0. Procedure A: 0.3 wt% bentonite followed by 2.67 mg/L of flocculant. Procedure B: 0.6 wt% bentonite and 5.34 mg/L of flocculant. Procedure C: 0.3 wt% bentonite and 5.34 mg/L of flocculant. Procedure D: 0.6 wt% bentonite and 2.67 mg/L of flocculant. An anionic synthetic aPAM (in a dual system with the same concentrations described in procedures A-D), was used as reference.

**Figure 10:**
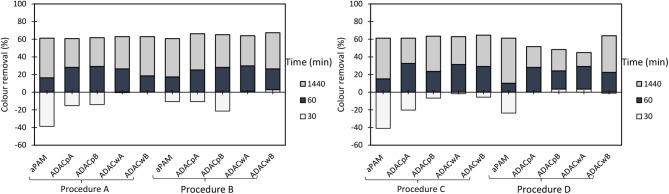
Turbidity removal of industrial effluent as a function of time of settling, using anionic cellulose-based flocculation agents from bleached pulp (ADAC_p_ A and B) and from wood chips (ADAC_w_ A and B), in a dual system with bentonite, at pH 7.0. Procedure A: 0.3 wt% bentonite followed by 2.67 mg/L of flocculant. Procedure B: 0.6 wt% bentonite and 5.34 mg/L of flocculant. Procedure C: 0.3 wt% bentonite and 5.34 mg/L of flocculant. Procedure D: 0.6 wt% bentonite and 2.67 mg/L of flocculant. An anionic synthetic aPAM (in a dual system with the same concentrations described in procedures A-D), was used as reference.

Anionic cellulose-based flocculation agents hold negatively charged groups, which are able to establish strong interactions with oppositely charged particles which can also be dispersed in the effluent. The developed anionic cellulose-based flocculants (ADAC_p_ and ADAC_w_ series) were tested in turbidity reduction of the industrial colored effluent. The variation of supernatant water turbidity, over time (30 min, 1 h, and 24 h), was used to evaluate the PELs performance in the treatment of this industrial effluent. The single system (only the anionic flocculant as described previously, see Figure 2), as well as a dual system with inorganic complexation agent (bentonite), were tested. The summary of the results while applying the dual systems are presented in Figures 8–10, for different pHs (1.5, 3.0, and 7.0).

Considering the poor results obtained only with the negatively charged flocculants, as it was expected since the effluent is, on average, negatively charged, the decision was to introduce a second component, a complexation agent (bentonite), with the objective to improve the flocculation kinetics. Figures 8–10 show the results obtained at pH 1.5, 3.0, and 7.0 (the initial effluent pH was 12.0) while using two different concentrations of flocculation agent (either anionic natural-based ADAC or synthetic aPAM), 2.67 mg/L (procedures A and D) or 5.34 mg/L (procedures B and C) and two different amounts of bentonite 0.3 wt% (procedures A and C) or 0.6 wt% (procedures B and D). For all the conditions tested, A-D, and tried pH levels (1.5, 3.0, and 7.0), better color removals were obtained after 24 h of treatment, when using the dual system bentonite-anionic PEL, compared to the single system, only with polyelectrolyte (Figure 2), at the same pH. As mentioned before for the cationic polyelectrolytes, also with anionic polyelectrolytes, the best results, after 24 h, were obtained when the pH was adjusted to 1.5, due to the lower effluent stability. In addition, bentonite positive charge increases with the increase of acidity level (Kim, 2003), which can significantly influence the interactions with the negatively charged effluent. Furthermore, in a dual system with bentonite, results obtained with the natural-based anionic flocculants are always better when compared to the results obtained with the synthetic aPAM reference. After adding bentonite, the effluent is destabilized and, in some cases, this can lead, initially, to some increase in turbidity of the system.

In general, the slightly positively charged bentonite surface (for acidic conditions) interacts with the negatively charged system (effluent), and further addition of the polymer allows faster and more effective flocculation, due to the charge neutralization and bridging mechanisms, resulting in rapid settling. In all tested procedures, at different pH levels, the same flocculation mechanisms must have been involved. However, with the increase of alkalinity, bentonite surface becomes less and less positively charged (Kim, 2003), the system becomes more stable, and less turbidity removal was obtained. Interactions between anionic cellulosic PEL and bentonite-dye complex should be mainly through the sulfonate groups (electrostatic) or hydroxyl groups (hydrogen bonding) of the cellulosic structure (Figure 1), although no favorable electrostatic interaction is anticipated directly between the anionic PEL and the negatively charged dye. Additionally, as can be seen, in Figures 8–10, considering the relatively high negative charge of the system, better clarification results were obtained at higher dosages of bentonite (procedures B and D).

Additionally, at the best treatment conditions (pH and dosages of bentonite and flocculant) ADAC_w_A and ADAC_w_B presented superior efficiency in the reduction of turbidity, especially for low contact times.

Comparing the performance of the treatments using ADACs with the treatments using cationic polymers (CDACs), combined, in both cases, with bentonite, the second ones performed always better (see Figure S2). This agrees with the average negative charge of the effluent, even if positive bentonite is added to the system. This is evident not only when we look at the final color removal after 24 h, but also analyzing the settling rate of the effluent particles, which is faster when using the cationic polymers.

**Figure 11:**
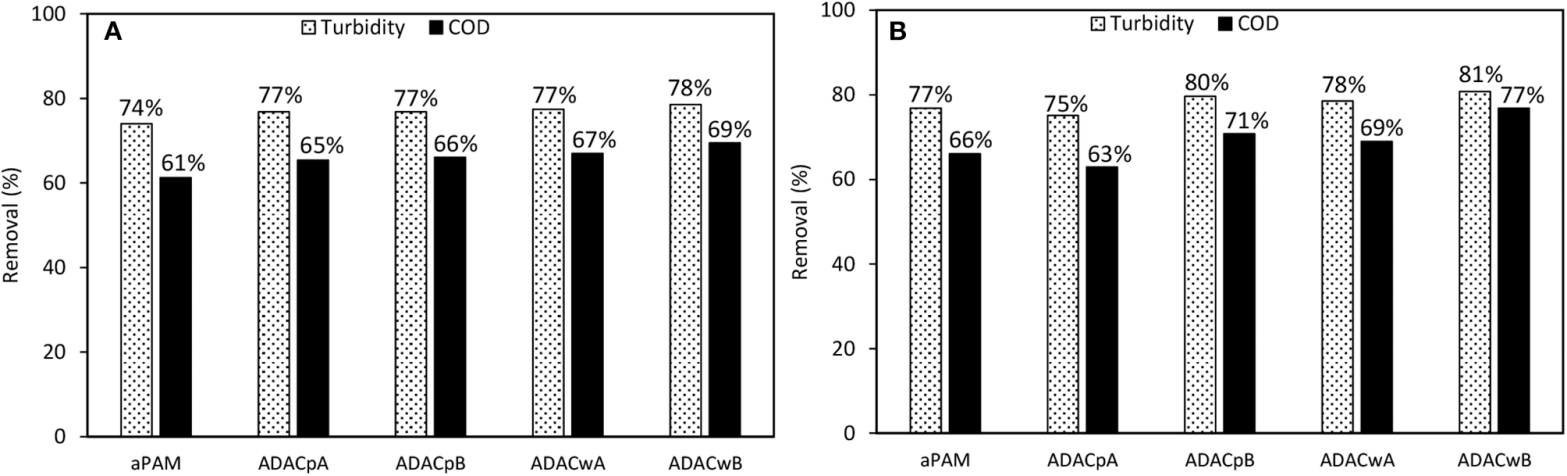
COD reduction and turbidity removal during the industrial effluent treatment with ADAC_p_A, B and ADAC_w_ A, B and reference aPAM, at pH 3.0, after 24 h of treatment, in two conditions: **(A)** 0.3 wt% bentonite followed by 2.67 mg/L of flocculation agent and **(B)** 0.6 wt% bentonite followed by 5.34 mg/L of flocculation agent.

For the anionic natural-based PELs obtained from modified bleached pulp, ADAC_p_A, obtained after 24 h of sulfonation, and ADAC_p_B, obtained after 72 h of reaction, for the anionic PELs derived from the pulp with the high lignin content, ADAC_w_A (24 h reaction) and ADAC_w_B (72 h reaction), and for the reference polymer aPAM, COD reduction efficiency was evaluated at pH 3.0, for two conditions: with lower dosages of bentonite and flocculant, which corresponds to procedure A (0.3 wt% bentonite followed by 2.67 mg/L of flocculation agent), and with maximum dosages of the two components, procedure B (0.6 wt% bentonite followed by 5.34 mg/L of flocculation agent). In Figure 11 we have plotted both the COD reduction after 24 h of treatment and the corresponding turbidity reduction. Once again, as it was observed for the cationic PELs (Figure 7), the COD reduction was closely related with the turbidity removal after 24 h. With the increase of effluent clarification higher COD reduction was observed. For the procedure with low addition of bentonite and flocculant components (procedure A), the COD reduction obtained was in the range of 65–69% for the new anionic natural-based flocculation agents produced (for CDACs it was 78–81%), while for the synthetic aPAM reference it was 61%. ADACs show lower performance in turbidity reduction, as already described, and also in COD reduction. Moreover, procedure B, with double levels of added components when compared to procedure A, presents slightly higher turbidity reductions, which results also in higher values of COD reduction, in the range of 69–77%, while for the synthetic reference 66% of COD reduction was achieved. Considering the overall results, natural-based flocculation agent ADAC_w_B, produced from the more heterogeneous fibers, Cw, seems to be the anionic cellulose-based flocculant that has the best performance in the treatment of the tested effluent, higher turbidity removal, with low dosage, and high COD reduction, for the described operation conditions at pH 3.

In general, a superior performance in turbidity reduction and water clarification was exhibited by the dual system with bentonite and cationic cellulose-based flocculation agents, compared to the dual system bentonite/anionic polyelectrolyte, due to the effluent nature, and repulsion interactions when using polyelectrolytes of similar charge to the average charge of the wastewater. Additionally, bentonite successfully destabilized the system, through the interaction with effluent impurities (especially dye molecules) allowing to create larger particles/larger complexes, heavier and easier to flocculate and settle.

Furthermore, natural-based PELs presented, in general, better turbidity and COD reductions, compared to the synthetic references. It is worth noticing that the use of procedure A with low dosages of bentonite (0.3 wt%) and natural-based PEL (2.67 mg/L), at pH 3.0, showed quite good turbidity reduction. Acidic conditions proved to be required to achieve adequate color removal.

#### Flocculation Monitoring by LDS

LDS was recently, successfully used in the flocculation monitoring of a real oily effluent (Lourenço et al., 2018) using especially designed synthetic flocculants, and following previous studies where only model particles were tested. Here, cationic natural-based PELs, CDAC_p_A, B, and CDAC_w_A, B, and anionic cellulose-based PELs, ADAC_p_A, B, and ADAC_w_A, B, with different features, were applied in the flocculation of an industrial colored effluent in dual system with bentonite and flocculation was monitored by LDS. Due to the different features of the tested cationic (CDACs) and anionic (ADACs) flocculants, differences in the flocculation process are expected to be observed. The conditions used in the LDS trials of the industrial colored effluent were based on the jar-tests experiments developed and described previously.

An example of the particle size distribution of the initial industrial colored effluent, at pH 3.0, after addition of the bentonite and at the end of the flocculation process (20 min of treatment), is shown in Figure S3, for the process carried out using cellulose-based flocculants, either the cationic CDAC_p_A or anionic ADAC_p_A. In both cases, the particle size distribution evolves from a bimodal distribution in the low particle size range to monomodal distributions which are displaced, with time, toward higher particle sizes, as expected. Moreover, ADAC_p_A showed slightly larger aggregates than CDAC_p_A. The median size of the particles in the initial industrial effluent, measured by LDS, was 0.106 μm, while after the addition of bentonite it was 3.88 μm, corresponding to the formation of effluent-bentonite complexes. The addition of flocculant, either cationic or anionic (4.0 mg/L), increased drastically the median size of the particles at the end of the process, monitored over 20 min. Adding CDAC_p_A, increased the median particle size to a value of 94 μm, while adding ADAC_p_A increased the median particle size to a value of 193 μm.

**Figure 12:**
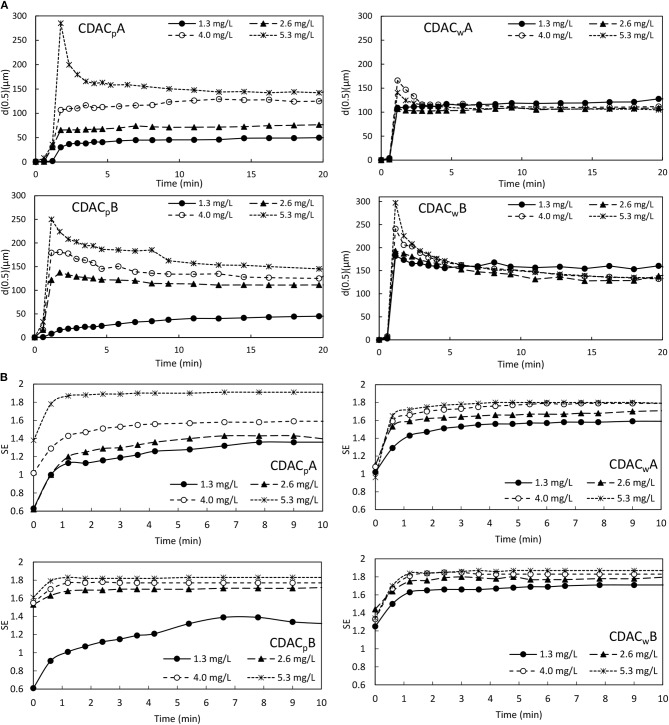
Industrial effluent treatment - evolution of **(A)** median particle size (d(0.5)) and **(B)** floc structure (SE) over time, obtained *via* LDS, for four different flocculant dosages of cationic cellulose-based polyelectrolytes obtained from bleached pulp, CDAC_p_A, B and obtained from pulp with kappa number of 26.7, CDAC_w_A, B, combined with bentonite.

Figure 12A and Figure S4 show the evolution of aggregate size over time, obtained by LDS, for four tested PEL concentrations (1.3, 2.6, 4.0, and 5.3 mg/L), for the cationic cellulose-based polyelectrolytes tested (obtained from bleached pulp, with high substitution degree, CDAC_p_A and lower substitution degree, CDAC_p_B, and obtained from pulp with kappa number of 26.7, with higher substitution degree, CDAC_w_A and lower substitution degree, CDAC_w_B). In these figures both the evolution of the median of the floc size distribution d(0.5) (Figure 12A), and the 90% undersize percentile diameter d(0.9) (Figure S4), are plotted. The trends in these curves are, in general, similar for all the cases tested if PELs produced from the same type of raw material are compared (CDAC_p_A with CDAC_p_B and CDAC_w_A with CDAC_w_B). Furthermore, the floc size reaches its maximum within 60 s after addition of flocculant to the effluent/bentonite system and then stabilizes. Figure 12A and Figure S4 demonstrate that the characteristics of used bio-PELs influence both aggregate size and the flocculation kinetics. Following the flocs size evolution with time, it is possible to conclude that in first minutes of the flocculation, aggregation predominates, allowing to obtain large floc size. After reaching the maximum value of floc size, breakage of the large flocs, even in low turbulent environment can happen (Rasteiro et al., 2008, 2011), and aggregates size stabilizes only when the balance between breakage and reaggregation occurs. Similar trends can be observed when analyzing both the evolution of the d(0.5) and d(0.9) with time.

The rearrangement of aggregates appears in all the tests while working at high polyelectrolyte concentrations (4.0 and 5.3 mg/L), especially when using PELs with lower charge density, CDAC_p_B and CDAC_w_B, which do also present higher molecular weight (considering the hydrodynamic diameter). This tendency is more obvious when looking at the d(0.9) plots, which correspond to the larger aggregates in the system. Additionally, with the increase of cationic-PEL dosage used in the effluent-bentonite system an increase in the floc size was observed. For all tested cellulose-based flocculants from bleached pulp, the influence of concentration is significant and is more pronounced for PEL with lower substitution degrees (CDAC_p_B). For the PELs produced from the pulp with a high kappa number of 26.7 (CDAC_w_A and CDAC_w_B) the influence of concentration is not so evident. This can be attributed to the expected wider distribution of molecular weight of these PELs, considering their higher heterogeneity (presence of other polymers besides cellulose). Additionally, the high charge and substitution degree of CDAC_p_A led to superior results regarding the flocs growth, over the slightly lower charged CDAC_p_B, which led to smaller aggregates, especially while working at lower polymer concentrations of 1.3 and 2.6 mg/L. This can be attributed to insufficient bridging of the particles while working with a lower charged polymer and lower concentrations. The predominant flocculation mechanism for the CDAC_p_ must be bridging, which explains why working at significantly low polymer concentrations results in small flocs, since only few particles will be bridged together in this case. It is also important to note, that the results obtained in the jar-test are in agreement to those obtained by LDS.

The SE profiles, calculated from the scattering matrix obtained via LDS, for the four cationic cellulose-based PELs, with four concentrations, were plotted in Figure 12B. For all the tests, independently of used cationic natural-PEL and concentration, the scattering exponent increases rapidly at the beginning of the flocculation process, when a rapid growth of the flocs size also occurs. The higher is the value of SE the more compact aggregates were obtained. Due to the very low particles size of the initial model effluent, it was not possible to obtain their SE value. The initial SE values in Figure 12B correspond to the effluent-bentonite system, and the continuous growth of SE indicates the continuous increase of floc compactness through the flocculation process. Comparing with literature (Lourenço et al., 2018), where an industrial oily-based effluent was flocculated with synthetic anionic polyelectrolytes, also under low turbulent conditions, SE values below 1.7 were reached for that case, while for the present industrial colored effluent-bentonite system, under similar stirring conditions, higher SE values were obtained in several trials, corresponding to more compact flocs. The floc structure results based on SE (Figure 12B) indicate that the flocculation process takes place by the bridging mechanism; supported by the fast flocculation rate and the relatively open aggregates (maximum SE around 1.9). Higher PEL concentration leads to more compact flocs, which correspond to higher values of SE, due to inclusion of more particles in the aggregate. In general, cationic flocculants from pulp with high lignin content (CDAC_w_A and CDAC_w_B) exhibit a lower influence of PEL concentration on floc compactness, agreeing with the trends observed when analyzing the influence of PEL concentration on floc size. Also, the flocs obtained with these two PELs are, in general, more compact than the ones obtained with CDAC_p_, when comparing results for the lower concentrations used. This may, again, be related to the wider distribution of molecular weights of these bio-PELs.

With the increase in charge density the less extended conformation of the polymer chain on the particles surface leads to more compact flocs, as it was also reported in the literature (Biggs et al., 2000). Comparing all the cationic polymers, the more compact flocs correspond to the situations where larger flocs were obtained, which is related to a more efficient flocculation process resulting in aggregation of more particles.

**Figure 13:**
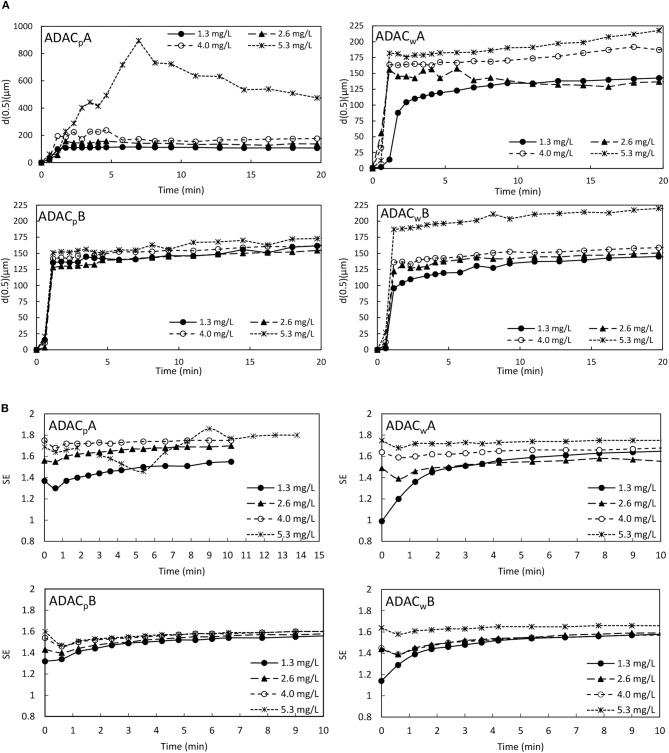
**(A)** Industrial effluent treatment - evolution of **(A)** median particle size (d(0.5)) and **(B)** floc structure (SE) over time, obtained *via* LDS, for four different flocculant dosages of anionic cellulose-based polyelectrolytes obtained from bleached pulp, ADAC_p_A, B and obtained from pulp with kappa number of 26.7, ADAC_w_A, B, combined with bentonite.

As it was observed while performing the jar-tests, with the increase of acidity the industrial effluent color removal obtained with ADACs increased, due to the lower stability of the system. However, using LDS has some limitations regarding the pH levels that can be used, due to which the tests with the effluent treated with ADACs were performed at pH 3.0. For this pH the effluent showed to be negatively charged, and thus, applying slightly positively charged bentonite followed by the addition of the negatively charged cellulose based PEL, led to effective flocculation results. Figure 13A and Figure S5 show the evolution of aggregates size over time, monitored by LDS, for four tested PEL concentrations (1.3, 2.6, 4.0, and 5.3 mg/L), for the anionic cellulose-based polyelectrolytes tested (obtained from bleached pulp, after 24 h of sulfonation procedure, ADAC_p_A and after 72 h of sulfonation, ADAC_p_B, and obtained from pulp with kappa number of 26.7, after 24 h of sulfonation, ADAC_w_A and after 72 h of sulfonation, ADAC_w_B). In these figures both the evolution of the median of the floc size distribution d(0.5), and the 90% undersize percentile diameter d(0.9) of the aggregates size distribution, are plotted.

From Figure 13A and Figure S5 it is clear that the features of tested PELs affect either floc size or the flocculation kinetics. The floc size reaches its maximum within few seconds after addition of flocculant to the effluent-bentonite system and then stabilizes. The only exception is when using ADAC_p_A, significantly longer time having been required to achieve the stabilization stage for the concentration of 5.3 mg/L, especially for the largest aggregates size, d(0.9). This can be attributed to the fact that much larger flocs are obtained for this case, which requires a longer period to reach equilibrium between aggregation and breakage. This is the bio-PEL from the ADACs that must have the largest molecular weight, considering the values of the hydrodynamic diameters measured (see Table 2). Considering the floc size evolution with time, it is possible to conclude that in the first minutes of the flocculation process aggregation predominates, allowing to obtain large aggregates. After reaching the maximum floc size, floc breakage of the large flocs, even in low turbulent conditions can occur, as it was also observed in flocculation of this colored effluent with cationic PELs, previously described, and, after that, eventually, equilibrium between breakage and reaggregation is achieved, and the floc size stabilizes. The rearrangement of flocs is not significant when working with the anionic cellulose-based polyelectrolytes, in most tested conditions, except for the highest concentration of ADAC_p_A, which led to the largest flocs. Comparing the results using CDACs (Figure 12A and Figure S4) and ADACs (Figure 13A and Figure S5), due to the low flocs rearrangement in the tests with ADACs, the final floc size obtained with these polymers was in general larger, when compared to the CDACs. In general, with the increase of anionic PEL dosage used in the effluent-bentonite system, an increase in the floc size was observed. For all tested anionic natural-based flocculants the influence of concentration is significant and is more pronounced for PELs obtained from the pulp with higher heterogeneity (high lignin content). Moreover, the predominant flocculation mechanism for anionic-PELs must also be bridging, which explains why working at significantly low polymer concentrations results in smaller flocs, since fewer particles will be bridged together in this case. Besides, anionic PELs led to obtain stronger aggregates with lower tendency to rearrange, when compared with the flocs obtained with the cationic bio-PELs.

Analyzing now the calculated SE profiles, obtained via LDS, for the four anionic bio-PELs, for the four concentrations tested, Figure 13B, the SE values are, in general, smaller than the ones obtained with the cationic bio-PELs (maximum value around 1.7). Thus, it is obvious that the anionic bio-PELs resulted in less compact flocs, when compared to the cationic cellulose-based PELs. Also, SE values vary less with time, when working with ADACs, what agrees with the lower rearrangement of the aggregates, as discussed when analyzing the flocculation kinetics.

The higher porosity of the flocs obtained with the ADACs can explain the lower turbidity reductions obtained in the jar-tests, with these polymers, since more porous flocs will take longer to settle.

The ADACs obtained from the pulp with high lignin content and higher heterogeneity resulted in slightly lower floc porosity (at the end of the flocculation process), especially for low concentrations, when compared with the PELs obtained from bleached pulp, for the same concentration. This must be the result of a higher affinity of the lignin, in the modified cellulose, to the effluent particles, especially at low concentrations, as was already described for jar-tests results.

In general, the success of the flocculation process and its kinetics is strongly dependent on the tested polyelectrolyte features and concentration, which conditions the flocculation mechanisms involved (Rasteiro et al., 2008). In the flocculation of the industrial effluent-bentonite system with both anionic and cationic, cellulose-based PELs, bridging is likely to be the predominant flocculation mechanism. Furthermore, in this effluent, the anionic cellulose-based flocculants, in general, tend to produce slightly larger aggregates with a lower tendency to reconform, but slightly less compact, when compared to cationic PELs. By using LDS, it was possible, for this effluent, to obtain information not only about the flocculation kinetics but also about the aggregates size distribution and their structure, as time evolved. One can conclude that LDS is a very useful technique to evaluate and differentiate polyelectrolytes for a desired application, not only in model effluents but also in industrial wastewaters.

## Conclusions

Cationic and anionic cellulose-based polyelectrolytes (PELs), varying in charge density, raw material used in the modification and chemical heterogeneity, were tested in terms of performance in the flocculation of an industrial colored effluent from a textile industry, revealing to be a very promising alternative to the conventional synthetic PELs, for the evaluated application. The experimental technique used, laser diffraction spectroscopy (LDS), which was tested in the present work for the first time in the monitoring of the treatment of an industrial colored effluent of very low particle size (effluent from textile industry), allowed obtaining data about the influence of the polyelectrolyte's characteristics and concentration on the flocculation process. Flocculation monitoring using LDS was complemented with traditional jar-tests, both techniques revealing the same trends, regarding the influence of the different parameters evaluated (flocculant type and concentration) on the flocculation process. Due to the very low particle size in the initial colored effluent, all flocculation tests were performed while applying a dual system with an inorganic complexation agent (bentonite).

Typically, polyelectrolyte bridging was involved in all tested flocculation systems either using cationic or anionic PELs. As expected, the results showed that with the increase of flocculant concentration, in general, the floc size increases and less porous aggregates are obtained, which is an indication of aggregation of a larger number of particles, agreeing with the trends observed previously in the jar-tests. Also, working with cationic PELs at higher concentrations favors the flocs rearrangement. Moreover, the effect of PEL concentration in the tested flocculation systems was more pronounced when using PELs from bleached pulp, probably due to their higher charge, and homogeneity, regarding molecular weight, when compared to the samples produced from the pulp with high kappa number (high lignin content, higher heterogeneity). In general, performance in flocculation was better when working in acidic conditions, for both the cationic and anionic bio-PELs. This is typical of flocculation processes applied to dye containing waste waters (Zemaitaitiene et al., 2003; Gao et al., 2007).

The use of LDS to monitor the flocculation processes allowed to determine some important flocs characteristics in a mild turbulent environment. It was possible to obtain information not only about the flocculation kinetics but also about the aggregates size distribution and their structure. Consequently, LDS proved to be a very useful technique to evaluate and differentiate polyelectrolytes for a desired application, in wastewater treatment, where important aspects are the knowledge of floc size and structure (large or small, open or compact) and flocculation kinetics, having in mind the final separation process to be used in the treatment (filtration, sedimentation, etc.).

Comparing the results obtained with the bio-PELs (color removal and COD reduction) with the ones obtained using standard polyacrylamides, better results were always obtained with the bio-PELs.

In conclusion, the results obtained show that the LDS technique can significantly improve the selection/optimization process of the adequate flocculant for a certain effluent treatment, before pilot-plant trials, after a primary, less extensive evaluation of performance in jar-tests, since it provides information about important characteristics of the flocs not available through the standard techniques.

## Data Availability Statement

The datasets generated for this study are available on request to the corresponding author.

## Author Contributions

KG performed the experiments and wrote the manuscript. KG and MR analyzed and interpreted the data. JG, JA, and OC contributed to the manuscript by commenting on the research findings and on the manuscript. All authors contributed to manuscript preparation, revision, read and approved the submitted version.

### Conflict of Interest

JA was employed by company AQUA+Tech. The remaining authors declare that the research was conducted in the absence of any commercial or financial relationships that could be construed as a potential conflict of interest.
