# Supplementary material for: Evaluation of Anionic and Cationic Pulp-Based Flocculants With Diverse Lignin Contents for Application in Effluent Treatment From the Textile Industry: Flocculation Monitoring
Source: Front Chem. 2020 Jan 30;8:5. doi: 10.3389/fchem.2020.00005 (PMC7002540; doi:10.3389/fchem.2020.00005)
Supplement: Supplementary file 1 [file Table_1.DOCX]

Evaluation of Anionic and Cationic Pulp-based Flocculants with Diverse Lignin Contents for Application in Effluent Treatment from the Textile Industry: Flocculation Monitoring

Kinga Grenda^1,2^, José A. F. Gamelas^1^, Julien Arnold^2^, Olivier J. Cayre^3^, Maria G. Rasteiro^1^*

***Supplementary Material***

Table S1. Chemical characterization of raw materials used for synthesis of dialdehyde cellulose.

| **Name** | **Kappa number** | **Cellulose content (wt%)** | **Xylan content (wt%)** | **Total lignin content (wt%)** |
| --- | --- | --- | --- | --- |
| **Commercially available *Eucalyptus* bleached kraft pulp^a^** | | | | |
| C_p_ | - | 85 | 14 | <0.1 |
| ***Eucalyptus* wood wastes pulp^b^** | | | | |
| C_w_ | 26.7 | 71.6 | 18.3 | 4.4 |

(a) values taken from the literature (Gamelas *et al.*, 2012); (b) xylan content corresponds to the hemicellulose fraction and does not include substituent acid groups

Table S2. Characteristics of the industrial coloured effluent sample.

| **Parameter** | **Industrial coloured wastewater** |
| --- | --- |
| pH | 12.0 |
| COD (gO_2_/L) | 1.475 |
| Turbidity (NTU) | 177 |
| ζ-potential (mV) | -52±4 |

Figure S1. Zeta potential of the coloured industrial effluent as a function of pH. The initial zeta potential of the industrial effluent without pH adjustment is highlighted.

Figure S2. Example of initial industrial effluent -Turquoise Blue and treated effluent after 24h of settling time, with cellulose-based flocculation agents from bleached pulp (cationic CDAC_p_A and anionic ADAC_p_A), in a dual system with bentonite, at pH 1.5; 3.0 and 7.0, when applying procedure A: 0.3 wt% bentonite followed by 2.67 mg/L of flocculant.

Figure S3. Particle size distribution of the industrial coloured effluent, at pH 3.0. Initial industrial effluent; effluent with addition of 0.15 wt% bentonite; effluent with further addition of 4.0 mg/L of the cellulose-based flocculants, either cationic CDAC_p_A or anionic ADAC_p_A, at the end of the flocculation (20 min).

Figure S4. Industrial effluent treatment - evolution of the 90% undersize percentile diameter of the particle size distribution, d(0.9), over time, obtained *via* LDS, for four different flocculant dosages of cationic cellulose-based polyelectrolytes obtained from bleached pulp, CDAC_p_A, B and obtained from pulp with kappa number of 26.7, CDAC_w_A, B, combined with bentonite.

Figure S5. Industrial effluent treatment - evolution of the d(0.9) over time, obtained *via* LDS for four different flocculant dosages of anionic cellulose-based polyelectrolytes obtained from bleached pulp, ADAC_p_A, B and obtained from pulp with kappa number of 26.7, ADAC_w_A, B, combined with bentonite.
